# Terrorist Attacks against Concerts and Festivals: A Review of 146 Incidents in the Global Terrorism Database

**DOI:** 10.1017/S1049023X22002382

**Published:** 2023-02

**Authors:** Harald De Cauwer, Dennis G. Barten, Derrick Tin, Luc J. Mortelmans, Gregory R. Ciottone, Francis Somville

**Affiliations:** 1. Department of Neurology, Sint-Dimpna Regional Hospital, Geel, Belgium; Faculty of Medicine and Health Sciences, University of Antwerp, Wilrijk, Belgium; 2. Department of Emergency Medicine, VieCuri Medical Center, Venlo, the Netherlands; 3. Department of Emergency Medicine, Beth Israel Deaconess Medical Center, Boston, Massachusetts; Harvard Medical School, Boston, Massachusetts; 4. Center for Research and Education in Emergency Care, University of Leuven, Leuven, Belgium; REGEDIM, Free University Brussels, Belgium; Department of Emergency Medicine, ZNA Camp Stuivenberg, Antwerp, Belgium; 5. Director, BIDMC Disaster Medicine, Beth Israel Deaconess Medical Center; Associate Professor, Harvard Medical School, Boston, Massachusetts, USA; 6. Department of Emergency Medicine, Ziekenhuis Geel, Geel, Belgium; Faculty of Medicine and Health Sciences. University of Antwerp, Wilrijk, Belgium; Faculty of Medicine, University of Leuven, Leuven, Belgium; CREEC (Center for research and education in Emergency Care). University of Leuven, Leuven, Belgium

**Keywords:** concerts, Counter-Terrorism Medicine, festivals, mass gatherings, terrorism

## Abstract

**Background:**

Mass gatherings are vulnerable to terrorist attacks and are considered soft targets with potential to inflict high numbers of casualties. The objective of this study was to identify and characterize all documented terrorist attacks targeted at concerts and festivals reported to the Global Terrorism Database (GTD) over a 50-year period.

**Methods:**

The GTD was searched for all terrorist attacks against concerts and festivals that occurred world-wide from 1970 through 2019. Analyses were performed on temporal factors, location, target type, attack and weapon type, attacker type, and number of casualties or hostages. Ambiguous incidents were excluded if there was doubt about whether they were exclusively acts of terrorism. Chi-square tests were performed to evaluate trends over time and differences in attack types.

**Results:**

In total, 146 terrorist attacks were identified. In addition to musical concerts, festivals included religious, cultural, community, and food festivals. With 53 incidents, South Asia was the most heavily hit region of the world, followed by the Middle East & North Africa with 25 attacks. Bombings and explosions were the most common attack types. The attacks targeted attendees, pilgrims, politicians, or police/military members who secured the concerts and festivals.

**Conclusion:**

This analysis of the GTD, which identified terrorist attacks aimed at concerts and festivals over a 50-year period, demonstrates that the threat is significant, and not only in world regions where terrorism is more prevalent or local conflicts are going on. The findings of this study may help to create or enhance contingency plans.

## Introduction

Mass gatherings are vulnerable soft targets for terrorist attacks. Concerts and festivals cause an influx of people at a specific time into a specific space, resulting in three target groups: attendees and emergency first responders, being soft targets, and governmental/security personnel.^1,2^ Mass gatherings also often involve the use of mass transit systems, another vulnerable target of terrorism. Attendees could easily be targeted while traveling to the event site, during the event, as well as on their way home after the event.^3^

The objective of this study was to identify and characterize all documented terrorist attacks targeting concerts and festivals reported to the Global Terrorism Database (GTD) over a 50-year period.

## Methods

A database search of the GTD was performed by using the Preferred Reporting Items for Systematic Reviews and Meta-Analyses (PRISMA) standard.^4^ The GTD is an open-source database containing over 200,000 global terrorism incidents that occurred in the period from 1970-2019.^5^ The GTD is maintained by the National Consortium for the Study of Terrorism and Responses to Terrorism (START) at the University of Maryland (College Park, Maryland USA) and is part of the US Department of Homeland Security (Washington, DC USA) Center of Excellence.^6^

The GTD defines a terrorist attack as follows: *“the threatened or actual use of illegal force and violence by a non-state actor to attain a political, economic, religious, or social goal through fear, coercion, or intimidation.”* ^6^ To be considered for inclusion in the GTD, the following three attributes must all be present:

1. The incident must be intentional;
2. The incident must entail some level of violence or immediate threat of violence; and
3. The perpetrators of the incidents must be subnational actors.

Additionally, to be included in the database, two out of three of the following criteria must be present:

1. The act must be aimed at attaining a political, economic, religious, or social goal;
2. There must be evidence of an intention to coerce, intimidate, or convey some other message to a larger audience than the immediate victims; and/or
3. The action must be outside the context of legitimate warfare activities.

An extensive description of the origin and the data collection methodology can be found in the GTD codebook, which is available on the START website.^5,6^ The full dataset of the GTD was searched for terrorist attacks against concerts and festivals. Due to loss of data, incidents from 1993 are not present in the online database. The following search terms were applied in the database: “festival(s)” and “concert(s).” Incidents were included if the aim of the attack was to target the festival/concert attendees or those tasked with securing the site. Attacks during transportation of the attendees to or from the event were also included.

Duplicates were excluded. Each attack that takes place as part of coordinated attacks is listed separately in the GTD. Every scene of a coordinated attack is listed as an individual incident site; however, the number of victims is divided between the different attacks by the GTD. In this study, these were listed as one incident and the total number of fatalities and injured that day were reported. In this way, the number of victims per attack and the number of incidents are more accurate. Two other events were listed as both festival and concert and were assigned to only one event type.

Events with insufficient information to determine whether the festival or concert was the target were further explored using reviews of gray literature. If information remained insufficient, the attacks were subsequently excluded. Last, incidents coded as “Doubt Terrorism Proper” were also excluded. These are incidents in which there was doubt if they were exclusively terrorism.^6^

Data collected per incident included temporal and spatial factors; location (country, world region); type of festival (ie, religious, music, town, new year); intended target (attendees, security forces, others); attack and weapon type; perpetrator type; and number of casualties.

Each entry was reviewed manually by the lead researcher for inclusion or exclusion based on the incident description. All collected data were exported into Excel spreadsheets (Microsoft Corporation; Redmond, Washington USA) and analyzed descriptively. Chi-squared tests, applied to evaluate the trends of incidents over time and the differences in casualties, were conducted with a significance level of P <.05.

## Results

**Figure 1.**
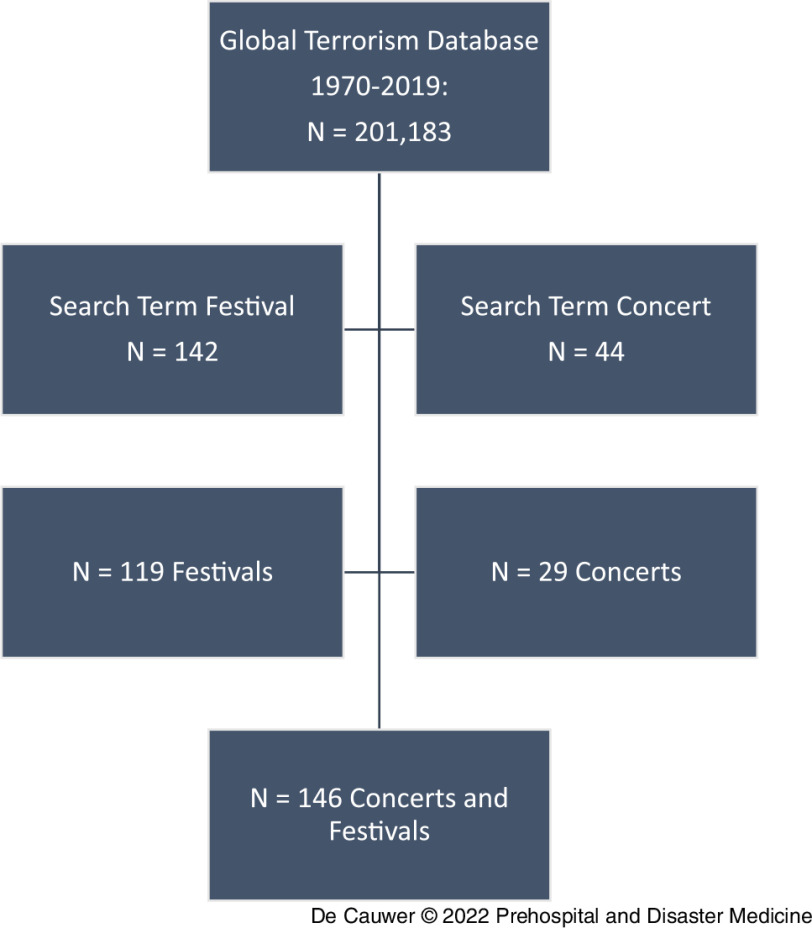
PRISMA Diagram. Step 1: *Identification* of all registered incidents in the GTD. Step 2: *Screening* for incidents with search terms “festival” and “concert.” Step 3: *Eligibility* – incidents excluded because of insufficient data, no festival or concert mentioned in data, or duplicates. Step 4: *Final Inclusion* with N = 2 events listed in both categories assigned to the most appropriate.

From 1970 through 2019, the GTD contained 146 incidents aimed at concerts and festivals that fulfilled the inclusion criteria (Figure 1). The attacks occurred in 37 countries and on five continents.

### Events per Year and Decade

**Figure 2.**
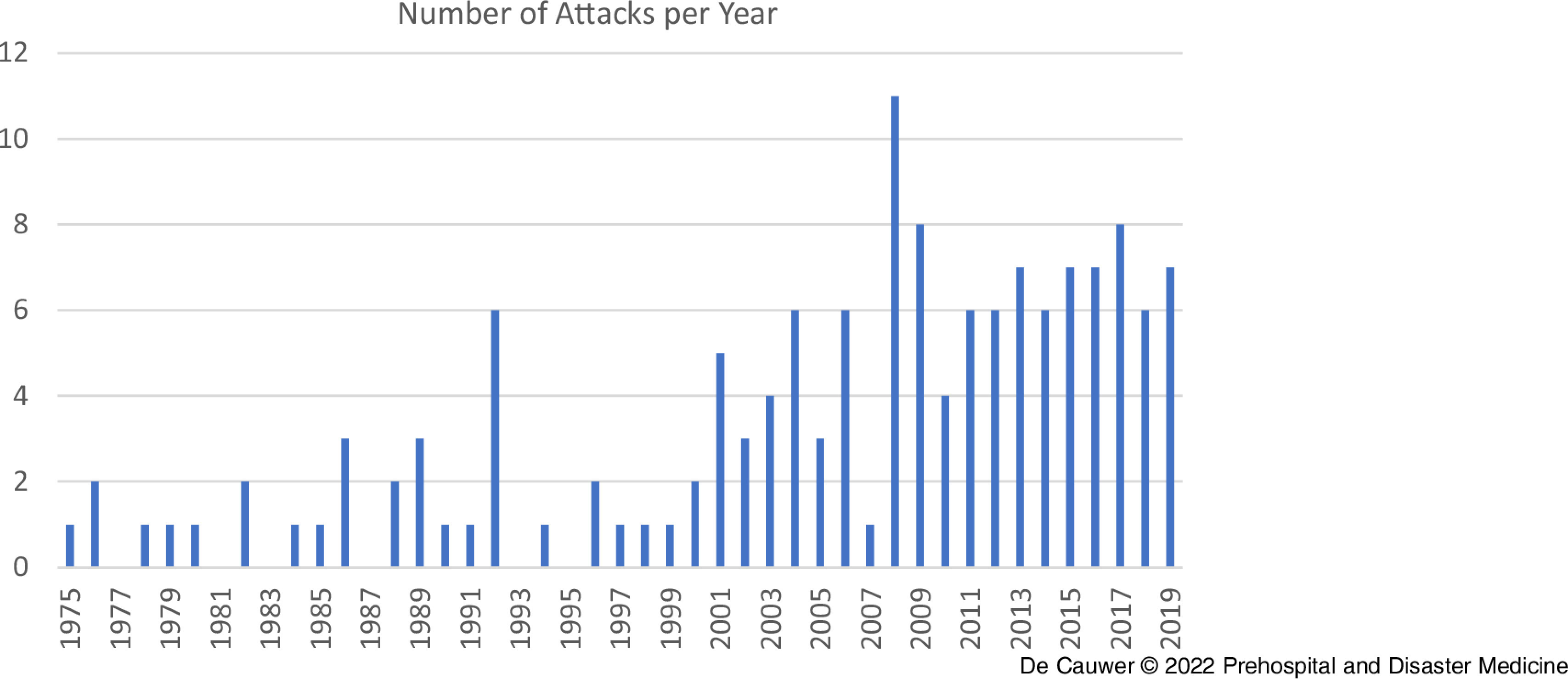
Number of Attacks per Year from 1970 through 2019.

**Figure 3.**
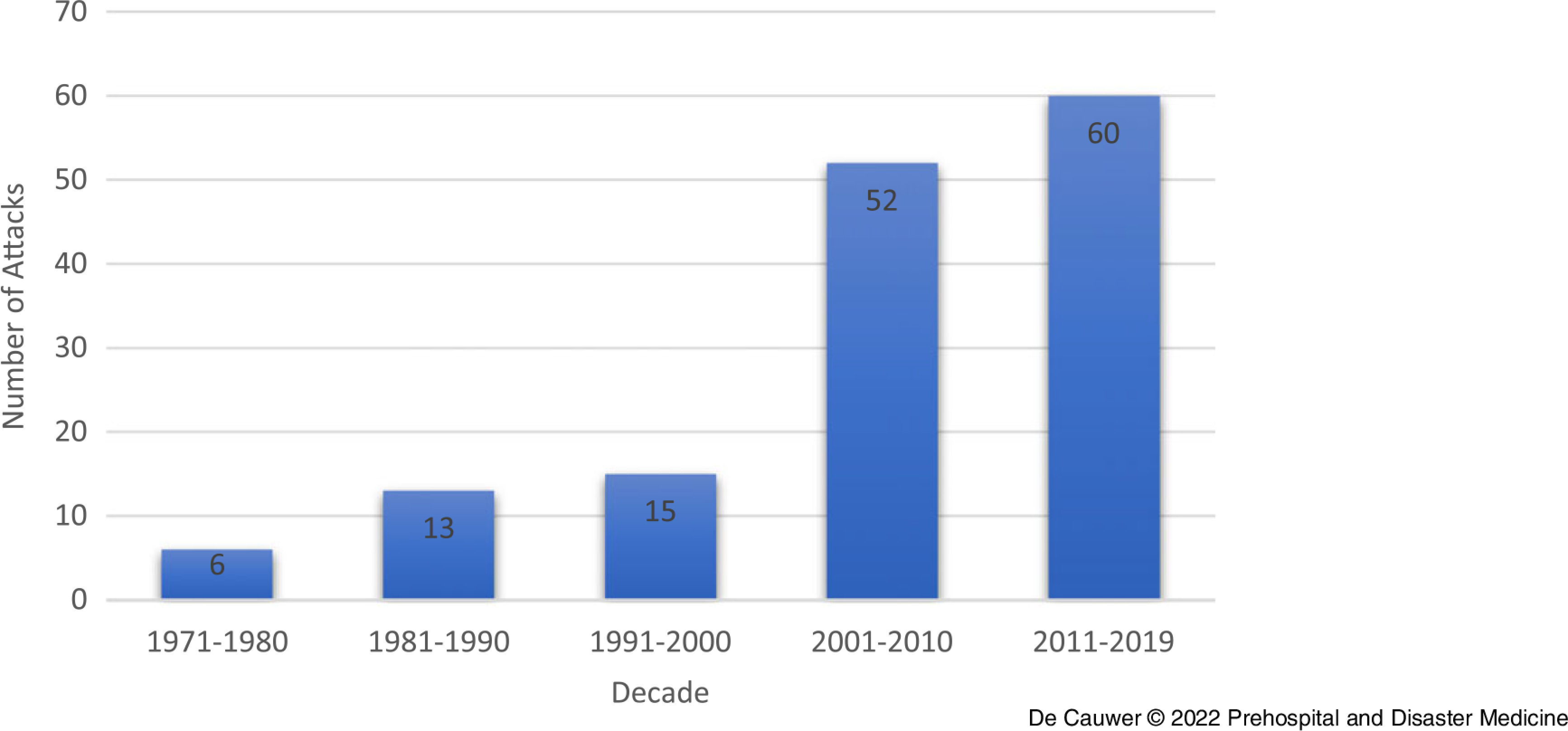
Number of Attacks per Decade.

Figure 2 and Figure 3 depict the number of terrorist attacks against concerts and festivals per year/decade. There was an increasing trend from 2000 onward. A chi-square test to evaluate the difference in the number of attacks per decade showed a significant difference in the number of attacks: *X* ^*2*^ *= 84.616*; *P* <.00001 (Appendix A; available online only).

### Events per Region

**Figure 4.**
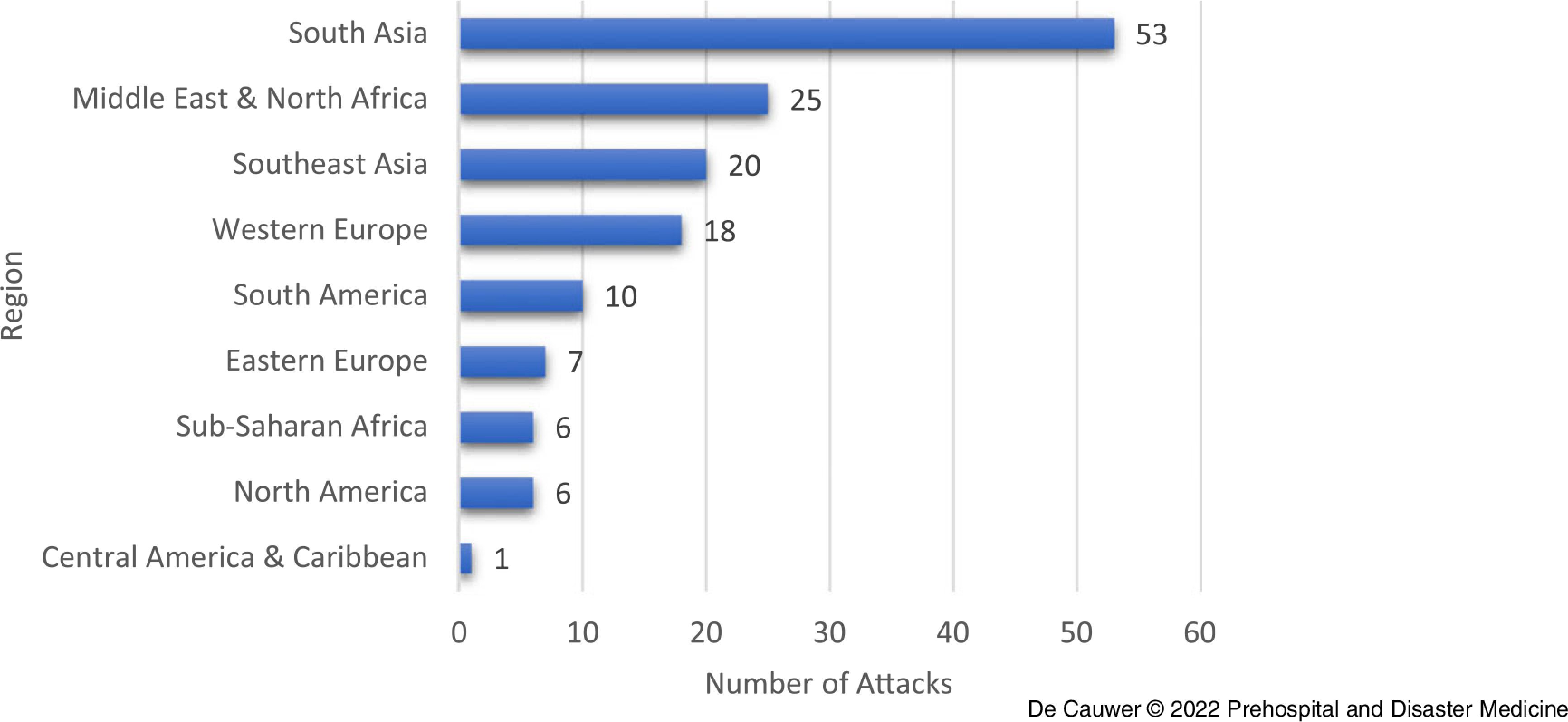
Distribution of Attacks per World Region.

With 53 (36.3%) out of 146 attacks, the most frequently affected region of the world was South Asia (Figure 4). Middle East & North Africa ranked second with 25 (17.1%) attacks, followed by Southeast Asia with 20 (13.7%) attacks and Western Europe with 18 (12.3%) attacks.

A chi-square test to evaluate the difference in number of attacks per continent showed a significant difference in number of attacks: *X* ^*2*^ *= 95.918*; *P* <.00001 (Appendix B; available online only).

India (n = 22; 15.1%), Iraq (n = 13; 8.9%), Pakistan (n = 12; 8.2%), and the Philippines (n = 11; 7.5%) were the most commonly affected countries.

### Attack Types and Weapon Types

**Figure 5.**
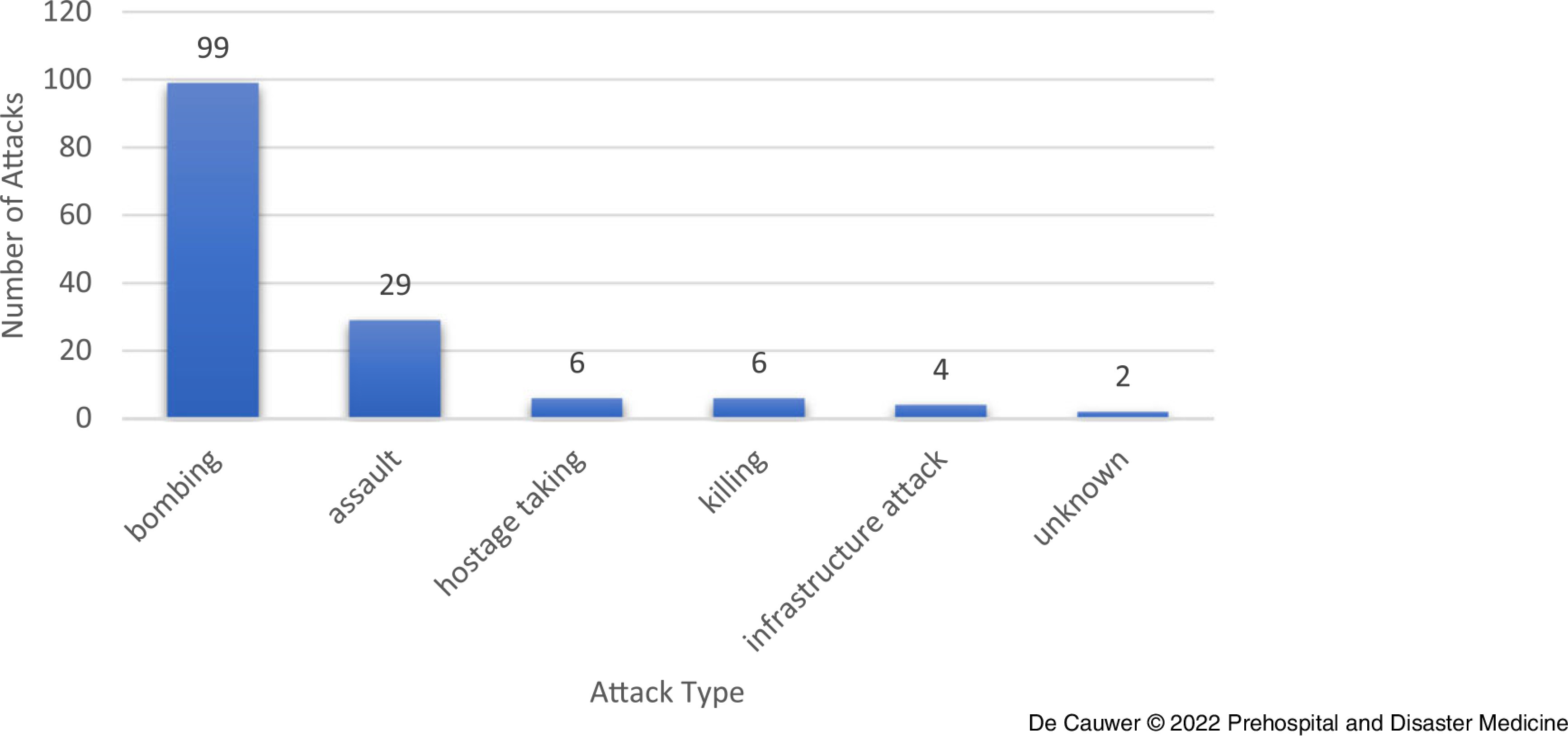
Attack Type of 146 Incidents Against Festivals/Concerts 1970-2019.

Bombings and explosions were the most frequently identified attack type (n = 99; 67.8%), followed by armed or unarmed assaults (n = 29; 19.9%); Figure 5. Kidnappings, assassinations, and infrastructure attacks were less frequently reported. In two incidents, the attack type was not mentioned.

The predominant weapon types were explosives (n = 97; 66.4%), followed by firearms (n = 27; 18.5%); in eight (5.5%) incidents, multiple weapon types were used (eg, explosives and firearms); Table 1. Other weapon types were uncommon.

**Table 1.** Weapon Type Used in Attacks Against Festivals/Concerts 1970-2019

Weapon Type	No. of Incidents
Explosives	97
Firearms	27
Mixed Weapons	8
Incendiary	4
Melee	3
Others	3
Unknown	3
Vehicles	1

### Festival Types and Targets

**Figure 6.**
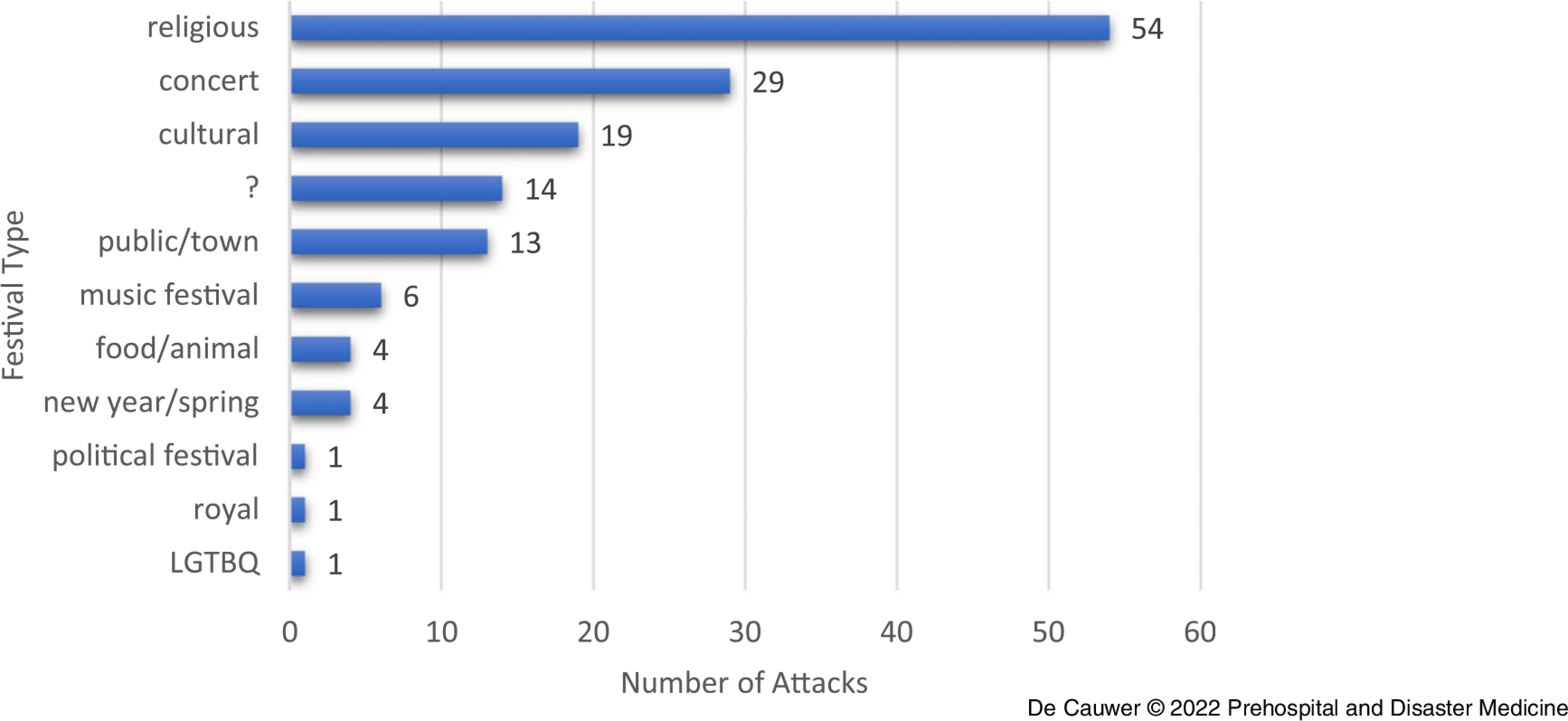
Different Festival Types Attacked during the Period 1970-2019.

Several types of festivals were targeted, with most being religious festivities (n = 54; 37.0%). The others are listed in Figure 6.

**Figure 7.**
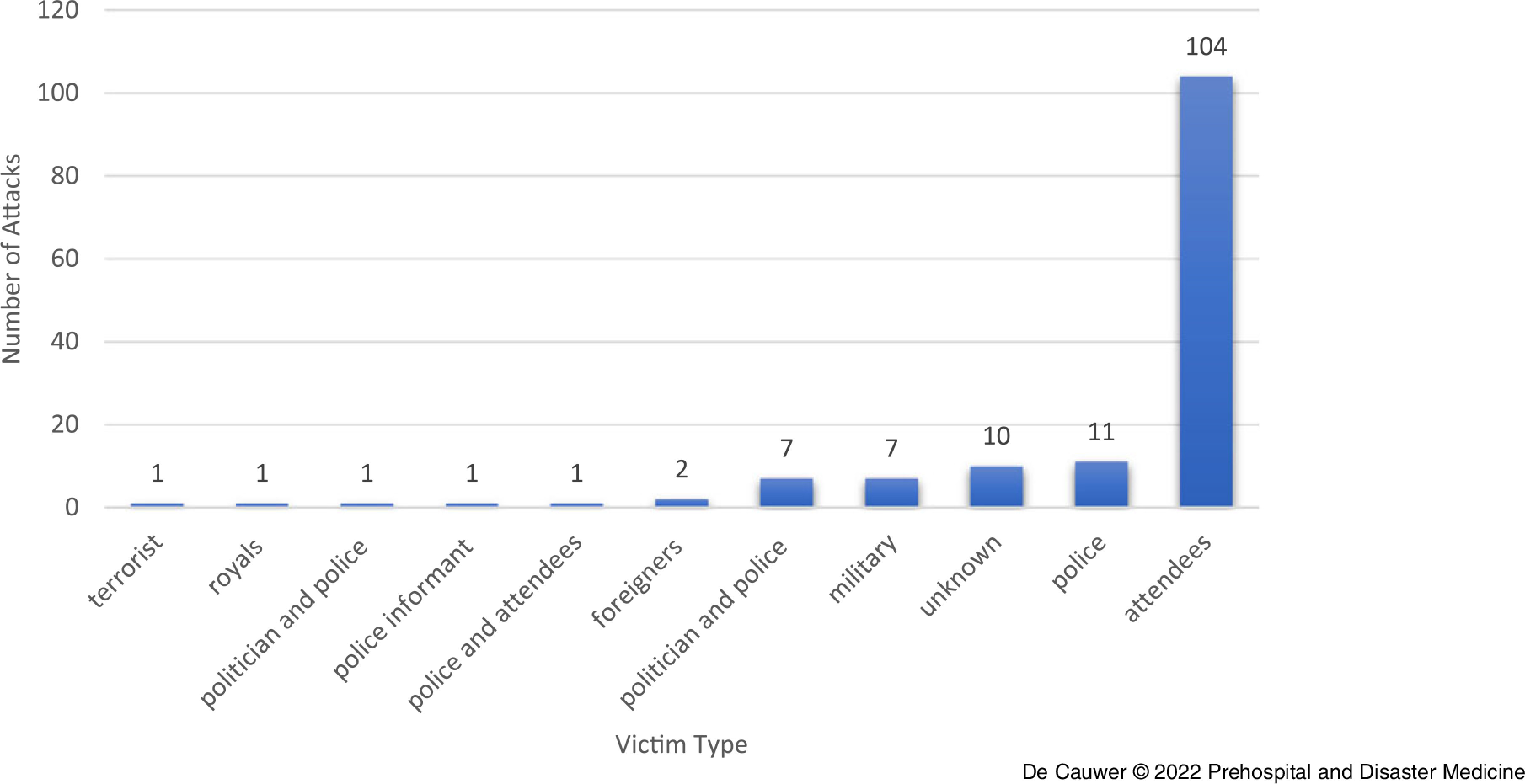
Target Type from List of 146 Incidents Against Festivals/Concerts 1970-2019.

In the vast majority of incidents, the attendees of festivals or concerts were the primary target (eg, pilgrims at religious festivals = 104 incidents; 71.2%); Figure 7.

In 13 incidents, houses of worship were attacked either at the entrance or inside: three were assaults and ten were bombings. In a series of coordinated attacks during the Persian New Year in Kabul, Afghanistan in 2019, a religious shrine and a hospital were targeted (bombing).

Security forces, deployed at mass gatherings, are the second largest group of targets (n = 20; 13.7%); Figure 7. Politicians attending festivals/concerts were targeted on eight (5.5%) of these occasions. In two incidents, foreigners (Niger in 2009 and Mali in 2009) were mentioned as a specific target. In one incident, a lone wolf targeted the royal family (The Netherlands in 2009). In another terror attack, children were targeted using an explosive device hidden in an ice cream vending car (Afghanistan in 2014).

### Number of Casualties per Region, Attack Type, and Target Type

In total, 802 confirmed fatalities (mean 5.6; median 1; minimum 0; maximum 100; SD = 14.6) were registered in the GTD (in three incidents, data were incomplete). A total of 3,439 people (mean 25.5; median 7; minimum 0; maximum 850; SD = 81.8) were injured. There were incomplete data in 11 incidents (Table 2).

**Table 2.** Number of Registered Casualties per World Region during Attacks Against Concerts and Festivals, 1970-2019

	People Killed (n)	People Injured (n)
South Asia	256	876
Middle East & North Africa	225	425
Western Europe	143	627
North America	68	947
Southeast Asia	43	281
Eastern Europe	42	189
South America	13	66
Sub-Saharan Africa	12	28
Central America & Caribbean	0	0
Australasia & Oceania	0	0
**Total**	**802**	**3439**

The deadliest attacks were bombings in Iraq in 2004 (100 fatalities, 267 injured) and in Pakistan in 2011 (52 fatalities, 102 injured); a shooting by an anti-government extremist at the Harvest Festival in the United States in 2017 (60 fatalities, 850 injured); the coordinated attacks and hostage taking in the Bataclan in Paris, France in 2015 (93 fatalities, 217 injured); and on Manchester Arena (United Kingdom in 2017, bombing) with 23 fatalities and 119 injured. In an assault in Iraq in 2008, 70 fatalities resulted with an unknown number of injured.

The regions with the most fatalities were South Asia, the Middle East & North Africa, and Western Europe (Table 2).

A chi-square test to evaluate the difference in the number of attacks per continent showed a significant difference in the number of attacks: *X* ^*2*^ *= 348.798*; *P* <.00001 (Appendix C; available online only).

Most injuries occurred in North America (the abovementioned attack on the Harvest Festival caused the majority of injured), followed by South Asia, Western Europe, and the Middle East & North Africa.

Figures for attack and weapon type show that bombings and explosions, followed by assaults with firearms, were the most devastating (Table 3).

**Table 3.** Number of Registered Casualties per Attack Type and Weapon Type during Attacks Against Concerts and Festivals, 1970-2019

Attack Type	People Killed (n)	People Injured (n)	Weapon Type	People Killed (n)	People Injured (n)
Bombing	434	2180	Explosives	442	2224
Assault	219	941	Firearms	202	923
Hostage Taking	103	225	Mixed Weapons	135	259
Assassination (Killing)	34	92	Unknown	13	1
Unknown	12	1	Vehicles	7	12
Infrastructure Attack	0	0	Incendiary	2	20
			Melee	1	0
			Others	0	0
**Total**	**802**	**3439**		**802**	**3439**

The number of casualties was the highest in terrorist attacks against religious concerts and festivals, followed by cultural festivals, public/town festivals, and food/animal festivals (Table 4).

**Table 4.** Number of Registered Casualties per Festival Type and Target Type during Attacks Against Concerts and Festivals, 1970-2019

Festival Type	People Killed (n)	People Injured (n)
Religious Festival	437	1044
Concert	244	1656
Public/Town Festival	42	115
Cultural Festival	21	183
Food/Animal Festival	17	274
New Year/Spring	12	51
Unknown	10	75
Music Festival	9	24
Royal Festival	7	12
LGBTQ Festival	3	5
Political Festival	0	0
**Total**	**802**	**3439**

Abbreviation: LGBTQ, Lesbian Gay Bisexual Transgender Questioning.

### Perpetrators and Number of Casualties per Perpetrator Type

Various perpetrator groups and related motives were mentioned in the GTD. However, in most of them, data were missing or perpetrators were not known. With some perpetrators, their motives and any relation to existing terrorist factions could not be demonstrated, while in some attacks, no one claimed responsibility. For those perpetrators the GTD did have enough information, separatists (n = 29; 19.9%), Islamic jihadists (n = 25; 17.1%), and communist factions (n = 14; 9.6%) were predominant.

Animal rights extremists were responsible for one attack on a bird festival (Italy in 1994). Far-right extremists conducted four terrorist attacks (United States in 1996, 2017, and 2019; Germany in 1980) listed in this series.

### Attacks at the Event Site versus in the Neighborhood

Attacks at the entrance and at the event site were compared to attacks during transportation. Most festival and concert fatalities and injuries occurred at the event site (Table 5).

**Table 5.** Number of Incidents, Fatalities, and People Injured per Incident Site

Incident Site	No. of Incidents	People Killed (n)	Mean Fatality	People Injured (n)	Mean Injured
At Event Site	101	710	7.0	3102	30.7
During Transportation	29	64	2.2	173	6.0
Unknown	16	28	1.7	164	10.2
**Total**	**146**	**802**	**5.5**	**3439**	**23.5**

## Discussion

Concerts and festivals are mass gatherings that attract many attendees and represent important cultural and religious events for surrounding communities.

This study assessed whether concerts and festivals represented an increased risk for terrorism. The GTD contains 146 terrorist attacks against concerts and festivals from 1970 through 2019, occurring in 37 countries and on five continents, most of which took place in South Asia and in the Middle East & North Africa. The attacks inflicted 3,439 injuries and 802 fatalities.

Though relatively rare, attacks on concerts and festivals can have significant psychosocial impacts on surrounding communities.

A recent study from Liang, et al on terrorist attacks against performing arts venues also contained incidents against music venues.^7^ However, Liang’s study, with the majority of incidents aimed at movie theatres and cinemas, assessed incidents with typically fewer numbers of attendees than this series. Comparatively, this study focused on larger mass gatherings, and also included religious festivals with very large crowds.

Since the 1970s, the number of terrorist incidents has grown globally, particularly in global regions where internal conflicts and/or sectarian violence exist and separatist groups are active. This may explain the large number of incidents and victims from attacks aimed at religious festivals, and the predominance of religious and separatist perpetrator groups involved.

North America and Europe were likewise hit by terrorist attacks. In contrast to the other continents, right-wing and Jihadi extremists, and so-called import-terrorism, accounted for most of the terrorist incidents.

In the early decades of the study period, only two far-right perpetrator groups conducted terrorist attacks (Army of God in the United States, and Wehrsportgruppe Hoffmann in Germany), while the communist groups or other far-left groups were predominantly active in the earlier decades. During the most recent decade, the surge of right-wing extremism, mixing racist motives, apocalyptic thinking, and conspiracy theories, experienced a boost in popularity with the coronavirus disease 2019 (COVID-19) pandemic.^8^ However, an increase in terrorist attacks linked to the COVID-19 pandemic will only be revealed later, when the data for the years 2020 through 2022 are available for research.

Attacks at the event site took more lives than during transportation to and from. During the event, more people are gathered in a closed space, making mass-casualty incidents more likely. The average number of fatalities and injuries per attack were comparable to those in an earlier study, with a mean of 2.2 fatalities and 5.2 injured per attack.^3^ During the event, the mean number of fatalities was three-times as high and the mean number of injuries was five-times as high.

To the best of the authors’ knowledge, this is the first peer-reviewed study to identify and characterize terrorist attacks against concerts and festivals using the GTD.

Another recent study on mortality at music festivals for the period 2016-2017 warned of an increase in mortality due to terrorism.^9^

In fact, this study, like others, particularly studied the effect of substance abuse and overdoses at music festivals and the emergency response, both prehospital at the event site, as well as in the emergency department of the nearby hospital.^9–11^

Some authors try to establish models to predict patient presentation rates and estimate the in-event health services requirements. These show some predictive value for similar events, but largely depend on other variables such as the age of attendees, indoor versus outdoor conditions, the length of the event, and even temperature.

The models had poor predictive value for other types of mass gatherings.^12,13^ Such models should also take into account the risk of terrorist incidents and the impact on prehospital health care. Contingency plans should consider auxiliary troops to be on standby and ready for deployment for both prehospital and intra-hospital care to manage the potential surge of victims. Disaster planners should foresee the burden of nearby hospitals, probably already challenged by additional patients (due to for example substance abuse), during a mass event, and the added burden posed by a terrorist incident, and in the worst case, a concomitant human stampede after an armed assault or bombing at a mass gathering.^9–11,14^

Because the police and military acting as security are potential targets, and health care workers are a growing target for terrorists, the location of prehospital health care posts should preferably be separated from security check points. At least two health care posts at distinct places should be made available.^8^

## Limitations

The GTD is the most comprehensive, up-to-date, open access and reliable database of terrorist incidents.^15^ The database, and therefore also this study, are however subject to several limitations. The data of events in the earlier decades are not complete. It is acknowledged by the GTD that at least in the first-half of the dataset, and in particular in the period from 1970 through 1989, the number of terrorist incidents is probably under-reported.^6,14^ The rise in the number of attacks since 2000 could be partly explained by this. The loss of data in 1993 has no significant role on the results of this series.

All information contained in the GTD reflects what is reported in multiple independent open access sources. Not all acts of violence and possible terrorist attacks may be covered by news media, and therefore, not all incidents may be included in the GTD. Only high-quality sources are used. This creates a possible selection bias, and is no guarantee as what the validity of the database information is concerned.^5,6^

Casualty numbers conflict across sources. Following the GTD protocol, the most recent reliable estimates are reported and used in this study. Furthermore, an under-estimation of the consequences of a terrorist incident might result from the GTD’s standard practice to report the lowest number of casualties when news articles provide conflicting information.^6^

Trends over time should be interpreted with caution because of these limitations.^6^ Conversely, the GTD is a key source for global data on terrorism incidents and is the best available database of its kind. It is evaluated as the most complete record of terrorist attacks in recent decades.^15^

Attempted but unsuccessful attacks are included in the GTD. However, threats, conspiracies, or the planning of attacks are not. The perpetrators literally had to be “out the door” to be included as an incident. In this series, perpetrators who were halted at check points or at the entrance of a festival or concert were included, although they did not cause victims.

State terrorism, although showing an increase in recent years, is not listed in the GTD.^8^

## Conclusion

This analysis of the GTD, which identified 146 terrorist attacks against concerts or festivals over a 50-year period, shows the variety in perpetrators and targets. Pilgrims attending religious festivals (soft targets) and security forces (state-related targets) were the primary targets of the GTD-registered incidents. The findings of this study may help to create or further improve contingency plans and preparedness efforts of prehospital health care and emergency departments.

## Supplementary material

For supplementary material accompanying this paper visit https://doi.org/10.1017/S1049023X22002382. click here to view supplementary material
