# Supplementary material for: Terrorist Attacks against Concerts and Festivals: A Review of 146 Incidents in the Global Terrorism Database
Source: Prehosp Disaster Med. 2022 Dec 21;38(1):33–40. doi: 10.1017/S1049023X22002382 (PMC9885432; doi:10.1017/S1049023X22002382)
Supplement: Supplementary file 1 [file S1049023X22002382sup001.docx]

**Appendix A:** Number of incidents against concerts and festivals per decade, 1970-2019

|  | Number of incidents | |  |  |
| --- | --- | --- | --- | --- |
|  | Observed | Expected | Chi-square value | *p-value* |
| 1970 - 1979 | 6 | 29.2 | 84.616 | p<.00001 |
| 1980 - 1989 | 13 | 29.2 |  |  |
| 1990 - 1999 | 15 | 29.2 |  |  |
| 2000 - 2009 | 52 | 29.2 |  |  |
| 2010 - 2019 | 60 | 29.2 |  |  |
| Total number of incidents | 146 |  |  |  |

The result is significant at p<.05

**Appendix B:** Number of incidents against concerts and festivals per continent, 1970-2019

|  | Number of incidents | |  |  |
| --- | --- | --- | --- | --- |
|  | Observed | Expected | Chi-square value | *p-value* |
| Europe | 25 | 29.2 | 95.918 | p<.00001 |
| Asia | 73 | 29.2 |  |  |
| Africa | 31 | 29.2 |  |  |
| North-America | 7 | 29.2 |  |  |
| South-America | 10 | 29.2 |  |  |
| Total number of incidents | 146 |  |  |  |

The result is significant at p<.05

**Appendix C:** Number of fatalities per continent, 1970-2019

|  | Number of incidents | |  |  |
| --- | --- | --- | --- | --- |
|  | Observed | Expected | Chi-square value | *p-value* |
| Europe | 185 | 160.4 | 348.798 | p<.00001 |
| Asia | 299 | 160.4 |  |  |
| Africa | 237 | 160.4 |  |  |
| North-America | 68 | 160.4 |  |  |
| South-America | 13 | 160.4 |  |  |
| Total number of incidents | 802 |  |  |  |

The result is significant at p<.05
